# Effect of Dietary L-Threonine and Toxin Binder on Performance, Blood Parameters, and Immune Response of Broilers Exposed to Aflatoxin B_1_

**DOI:** 10.3390/toxins14030192

**Published:** 2022-03-04

**Authors:** Aydin Mesgar, Habib Aghdam Shahryar, Christopher Anthony Bailey, Yahya Ebrahimnezhad, Anand Mohan

**Affiliations:** 1 Department of Animal Science, Shabestar Branch, Islamic Azad University, Shabestar 5381637181, Iran; aidin.msgar@gmail.com (A.M.); ha.shahryar@iaushab.ac.ir (H.A.S.); ebrahimnezhad@iaushab.ac.ir (Y.E.); 2 Department of Poultry Science, Texas A&M University, College Station, TX 77843, USA; 3 Department of Food Science and Technology, University of Georgia, Athens, GA 30602, USA

**Keywords:** aflatoxin, threonine, toxin binder, Mycofix Plus, broiler, performance, immunity

## Abstract

To evaluate the effect of L-Threonine (L-Thr) and Mycofix^®^ Plus (MP) on aflatoxicosis, an experiment with a 3-way ANOVA model was carried out with 8 replicates and 640 birds. Treatments included two levels of L-Thr (100% and 125% of the requirements, Cobb 500, Cobb-Vantress), Aflatoxin B_1_ (AFB_1_) (0, 500 ppb), and MP (0, 1 g/kg). As the main effects showed, AFB_1_ decreased breast meat yield and carcass percentage (*p* < 0.001), serum urea, antibody titer against infectious bronchitis virus (IBV), and bone density (*p* < 0.05), while it increased the plasma concentrations of glucose and alkaline phosphatase (ALP) (*p* < 0.05). Mycofix Plus improved the grower feed intake (FI), tibia fresh weight, and body weight (BW) to bone weight (*p* < 0.05). L-Threonine increased the grower FI, breast meat yield, serum aspartate transaminase (AST), and glutathione peroxidase (GPX) (*p* < 0.05). There were positive interactions with breast meat yield, cholesterol, lactate dehydrogenase (LDH), and IBV titer. Of the treatments used, the combination of L-Thr and MP without AFB_1_ improved breast meat and carcass percentage. L-Threonine and MP significantly improved IBV titer in birds challenged with AFB_1_ (*p* < 0.001). In conclusion, L-Thr and MP were beneficial to improve immunity.

## 1. Introduction

Mycotoxins are secondary metabolites produced by fungi that grow in hot, humid climates and are detrimental to poultry health and performance [1]. Among mycotoxins, depending on the region, aflatoxins are the primary concern in the poultry feed industry, and aflatoxin B_1_ (AFB_1_) is the most dangerous and common toxin in foodstuffs among aflatoxin G_1_ (AFG_1_), aflatoxin B_2_ (AFB_2_), and aflatoxin G_2_ (AFG_2_) [2]. Aflatoxins are mainly produced by *Aspergillus flavus* and *Aspergillus parasiticus* (*A. parasiticus*) [3], which commonly contaminate corn and other crops, from planting to harvesting and storage to processing [4].

It has been reported that aflatoxicosis negatively affected performance (40–1500 ppb) [3,4,5,6,7] and blood parameters (500 ppb) [3], disturbed the immunity (50–2000 ppb) [3,8,9,10,11], reduced the antioxidant capacity (100–2000 ppb) [9,12,13,14,15,16,17], increased the blood or tissue malondialdehyde (MDA) concentration (74–2000 ppb) [9,12,13,15,16,17,18], and damaged the intestinal morphology (100–2000 ppb) [12,19,20,21,22,23] and intestinal microbiota (40 ppb) [4,7] in broiler chickens. Moreover, previous studies represented an opposed relationship between bone mineralization and AFB_1_ (625–10,000 ppb) [24], or a negative correlation between the calcification or utilization of cholecalciferol and AFB_1_ (500–20,000 ppb) [25]. However, these calcification studies are rare. Aflatoxin-contaminated feeds threaten poultry health and performance and lead to economic losses by depressing meat production. According to FDA, 2019 [26], the upper limit with respect to adult poultry is 100 ppb. However, different concentrations are expected depending on the temperature, relative humidity, and storage conditions. In the United States, any cereal grain (feedstuff) containing over 1000 ppb must not be allowed to enter commerce (usually buried in the fields if discovered) where 500 ppb can be a practical testing concentration, as an occasional dose under inappropriate conditions.

The absorption rate of aflatoxins from the gastrointestinal tract is quick. Compared to other organs, the gastrointestinal tract is the first site to contact mycotoxins, which makes it more vulnerable to AFB_1_ [27]. Aflatoxin B_1_ alters intestinal morphology [19,21,22], which can reduce the absorption of nutrients. According to some reports, aflatoxin reduced the absorption of essential nutrients, and probably increased the amino acid requirements [28]. As Grenier and Applegate reported in 2013 [28], aflatoxins are absorbed by passive transport, and the absorption rate is more than 80 percent, regardless of the species. The gastrointestinal tract is the first line of contact with mycotoxins [27,28], and often at a higher concentration than other tissues, due to the high protein turnover and activated cells of the gut epithelium. Mycotoxins can disturb nutrient absorption, barrier function, or facilitate the persistence of intestinal pathogens and potentiate intestinal inflammation, and aflatoxin probably increases the amino acid requirements and disturbs the utilization of essential nutrients [28].

Agriopoulou et al. (2020) [29] noted numerous mycotoxin control strategies, including physical treatment (sorting, processing, storage, radiation, cold plasma, and toxin binders), chemical control (bases such as ammonia and hydrated oxide, chitosan, and ozone treatment), biological control (bacteria, yeast, food fermentation, and non-toxic strains of fungi), enzymatic detoxification, and novel strategies (nanoparticles and plant extracts) as post-harvest controls. Several of these approaches to mitigating the adverse effects of aflatoxicosis, such as additives containing adsorbents, probiotics, prebiotics, and phytogenics, are among the most practical, safe, and cost-effective methods. In this regard, there are three ways to manage mycotoxins, containing biological (probiotics and prebiotics), physical (adsorbents), or chemical (herbal essential oils) methods [3]. The multi-component toxin binder (Toxin Binder + Toxin Deactivator) applied in this study (Mycofix Plus MP, Biomin GmbH, Herzogenburg, Austria) is a combination of mineral adsorbents, specific enzymes, biological components (biotransformation), plants, and algae extracts (bio-protection). The high-quality bentonite (dioctahedral montmorillonite) in MP is a powerful binder with more than 90% binding affinity to aflatoxins, based on the European Union Reference Laboratory method [30]. However, there are not enough reports about the efficacy of MP in broilers fed low levels (Industry-Relevant) of aflatoxins.

L-Threonine (L-Thr) is often the third limiting amino acid in corn–soybean meal-based diets, and plays a vital role in many areas, including gut health, morphology, and function, the optimal utilization of total sulfur amino acids and lysine (Lys), immunity, carcass traits, the synthesis of structural proteins, antibody, uric acid, and pancreatic enzymes, the maintenance of intestinal barrier, and mucin synthesis [31]. The mucus layer protects the intestinal mucosa, which contains mucins, heavyweight glycoproteins that require L-Thr for the synthesis. The supplementation of L-Thr above the National Research Council (NRC, 1994) [32] requirements has been reported to be helpful for the gut health and immunity of broilers [31], and the best results on antioxidant function and gut morphology were observed at 125% of NRC, 1994 [32] recommendations [33].

The inclusion of excess L-Thr above NRC, 1994 [32] requirements has been repeatedly worked on, while new researches on the last commercial requirements are still needed under stress, or in abnormal conditions or diseases.

L-Threonine is an essential amino acid for poultry, and its influence on performance and intestinal function may reduce the harmful effects of AFB_1_ in birds.

Therefore, considering the potentials and capacities of L-Thr and MP, this research aimed to evaluate the efficacy of dietary L-Thr and MP, with or without 500 ppb of AFB_1_ (Aflatoxins, 718 ppb), as an occasional dose or at a low level [28,34].

## 2. Results

### 2.1. Performance and Carcass Traits

The treatments did not significantly affect the feed intake (FI), body weight gain (BWG), and feed conversion ratio (FCR) (Table 1 and Table 2). Nevertheless, a significant increase in FI was observed in the grower period. Treatment 7, including L-Thr and MP, resulted in the highest FI (548.9 g) compared to other treatments, and this difference was significant (*p* < 0.05) in contrast with T_1_, T_2_, T_3_, and T_5_ (544.4, 543.4, 542.7, and 543.7 g, respectively). As the main effects, L-Thr and MP increased the FI by 0.39% and 0.40%, respectively (*p* < 0.05). However, there were no significant effects on the total FI, BWG, FCR, European Production Efficiency Factor (EPEF), and European Broiler Index (EBI) (Table 3).

As noted in Table 4, T_7_ resulted in the highest percentage of breast meat (23.32) compared to other treatments, except control (22.75) (*p* < 0.01). The breast meat yield was significantly decreased by T_2_, T_3_, T_4_, and T_8_ compared to control (*p* < 0.01). As the main effect, AFB_1_ decreased the relative weight of breast meat by 1.53%, significantly (*p* < 0.001). L-Threonine increased breast meat yield by 0.9% (*p* < 0.05). The 2-way interaction effect between L-Thr and MP was positive and increased breast meat yield, in contrast with MP alone (*p* < 0.05) (data table is contained within the supplementary material; Table S1). The carcass percentage was significantly decreased by T_2_, T_3_, T_4_, T_6_, and T_8_ compared to control (*p* < 0.01). The best carcass yield was observed in T_7_ (62.38%), significantly higher than other treatments, except for the control. (61.61%) (*p* < 0.01). As the main effect, AFB_1_ decreased the carcass yield from 60.68% to 58.74% by 1.94% (*p* < 0.001). The other variables, such as wings, back, neck, and thigh (WBNT), drumsticks, liver, spleen, kidneys, bursa of Fabricius, pancreas, heart, gizzard, and abdominal fat, were not significantly affected by the treatments in this study (Table 4 and Table 5). However, AFB_1_ numerically decreased the relative weight of the drumsticks by 0.49% (*p* = 0.06).

### 2.2. Blood Biochemical Parameters and Serum Enzymatic Activity

The concentration of glucose, cholesterol, triglycerides, high-density lipoprotein (HDL), low-density lipoprotein (LDL), very low-density lipoprotein (VLDL), uric acid, urea, total protein, albumin, and globulin are presented in Table 6 and Table 7. As the main effect, AFB_1_ increased the serum glucose level by 9.11% (*p* < 0.05). A 2-way interaction between MP and AFB_1_ on cholesterol resulted in a higher concentration compared to AFB_1_ alone (*p* < 0.05) (data table is contained within the supplementary material; Table S2). Aflatoxin B_1_ decreased HDL concentration compared to the control in a 2-way interaction between MP and AFB_1_ (*p* < 0.05) (data table is contained within the Supplementary Material; Table S2).

As a 2-way interaction between L-Thr and MP, the supplementation of L-Thr resulted in a higher LDL in contrast with the control (*p* < 0.05) (data table is contained within the Supplementary Material; Table S1). As the main effect, AFB_1_ decreased the serum concentration of urea by 21.61% (*p* < 0.05).

The levels of aspartate transaminase (AST) and alkaline phosphatase (ALP) were significantly increased by L-Thr and AFB_1_ (10.21% and 5.92%), respectively (*p* < 0.05) (Table 8). Aflatoxin B_1_ increased the concentration of alanine aminotransferase (ALT) in a 2-way interaction with MP compared to the control (*p* < 0.05) (data table is contained within the supplementary material; Table S2). The 2-way interaction between MP and AFB_1_ on lactate dehydrogenase (LDH) was significant (*p* < 0.05), and MP decreased the concentration of LDH (data table is contained within the Supplementary Material; Table S2).

### 2.3. Stress Status, Antibody Titer and Antioxidant Capacity

All of the related variables are presented in Table 9 and Table 10. The treatments did not significantly affect the Heterophil (H), Lymphocyte (L), and H to L ratio. However, the lowest and highest percentages of H (32.08) and L (67.92) were observed in control, MP, and L-Thr plus MP treatments; also, the best ratio of H to L (0.47) was observed in the control group and birds fed supplemental MP alone.

As the main effect, AFB_1_ decreased the antibody titer against infectious bronchitis virus (IBV) almost by 0.1% (*p* < 0.05). The IBV titer was significantly lower in treatments containing AFB_1_ compared to control. The best concentration of IBV titer among the treatments was observed in T_8_ and the control. Antibody titer against IBV was significantly higher in T_8_ (3.833) compared to T_2_ (3.824), T_4_ (3.821), and T_6_ (3.818), showing the positive interaction between L-Thr and MP. The interaction effect of L-Thr and MP was significant (*p* < 0.01) and showed an improved titer against IBV via the supplementation of MP (data table is contained within the Supplementary Material; Table S3).

In addition, similar results were obtained for the interaction effect between MP and AFB_1_ (*p* < 0.01) when MP increased the antibody titer against IBV compared to the AFB_1_ group (data table is contained within the Supplementary Material; Table S4). The independent variables did not affect the infectious bursal disease virus (IBDV) titers significantly.

The serum concentration of glutathione peroxidase (GPX) significantly increased by 17.49% via the inclusion of L-Thr (*p* < 0.05). The highest and the lowest GPX concentrations were observed in T_7_ (L-Thr + MP) and T_2_ (AFB_1_), respectively. The serum concentrations of superoxide dismutase (SOD) and catalase (CAT) were not significantly affected by the treatments (Table 10).

### 2.4. Meat Quality and Tibia Characteristics

The meat quality variables such as pH, water holding capacity (WHC), cook loss, and MDA were not affected (data table is contained within the Supplementary Material; Table S5). As the main effect, MP increased tibia fresh weight by 0.03% (*p* < 0.05) and significantly improved body weight (BW) to bone weight by 4.51% (*p* < 0.05) (Table 11). There were no more significant effects on the other variables, except for bone density. As the main effect, AFB_1_ significantly decreased bone density by 3.33% (*p* < 0.05) (Table 12).

### 2.5. Intestinal Morphometry and Cecal Microflora

Jejunal indices, including villus height (VH), villus width (VW), crypt depth (CD), VH:CD, muscular layer, surface area, and apparent absorptive surface area were not significantly affected by the inclusion of L-Thr or MP (Table 13). The AFB_1_ did not significantly alter the jejunal morphometry; however, as the main effect, the muscular layer was numerically thinner in the AFB_1_ group (*p* = 0.06).

Total aerobic bacteria counts (TAC), *E. coli*, and *Lactobacilli* were not significantly affected in the present study. However, L-Thr at 125% of the recommended intake did numerically reduce the log_10_ of colony-forming units of cecal *E. coli* by 0.45 (CFU) g^−1^ in the presence of AFB_1_ (data table is contained within the Supplementary Material; Table S6).

## 3. Discussion

This study was designed to evaluate the efficacy of dietary L-Thr and MP to reduce the harmful effects of a commercially relevant concentration of aflatoxin (AFB_1_, 500 ppb) over the course of 5 weeks. Growth performance, carcass traits, blood–biochemical metabolites, enzymatic activities, immune response, serum antioxidant capacity, meat quality, tibia characteristics, intestinal morphometry, and cecal microflora were studied. The whole period performance was not affected by AFB_1_, L-Thr, or MP. Nevertheless, FI was increased by supplemental L-Thr in the grower period, which may relate to a triggered appetite-regulating mechanism [31]. Ahmed et al. (2020) [31] reported a better growth performance of Ross 308 broilers fed extra L-Thr above NRC; 1994 [32] recommended requirements (110 and 120%) due to more dietary L-Thr to support the growth and digestive system, followed by an enhanced apparent ileal digestibility of proteins and amino acids. However, whole period growth performance was not significantly affected by L-Thr in this study. Chen et al. (2017) [35] observed no effects of supplemental L-Thr (1 and 3 g/kg of feed) on the growth performance of Arbor-Acres Plus broilers for 21 days. Our findings are almost similar to Min et al. (2017) [33], who found no significant differences among L-Thr levels (100, 125, and 150% of NRC, 1994 requirements) on the growth performance of Arbor Acre broiler chickens from 0 to 21, 22 to 42, and 0 to 42 days of age. The appetite stimulation effect by MP in the grower period may relate to its phytogenic compounds, such as plant and algae extracts. However, similar results were not observed for other periods; more research is needed to make a conclusion. Moreover, a non-significant higher FCR was observed in MP treatment at finisher-2, and the reason is not readily apparent. Dänicke et al. (2003) [36] reported a tendency to stimulate the FI in Lohmann male broilers fed 2.5 g of MP/kg of diet. Their findings showed decreased final live BW, increased FCR, and impaired performance, regardless of mycotoxin concentration. From 1 to 28 days, no significant improvements in FI, BWG, and FCR were observed by 2 g of MP/kg of diet in Ross 308 male broiler chickens compared to control [37]. Moreover, Hanif et al. (2008) [38] observed no significant positive effects of MP (1 and 2 g/kg) on the FI, BW, and FCR of Starbro broilers over the course of six weeks compared to the control group; however, a higher MP level resulted in a higher BW in week 5, in contrast with the control group. Giambrone et al. (1985) [39] had reported that AFB_1_ less than 1000 ppb is subclinical for birds with a balanced diet and excellent management. Likewise, Chen et al. (2014) [34] showed that FI, weekly BWG, and feed efficiency were not affected by the 500 and 1000 ppb of AFB_1_; total BWG was lower than the control treatment at 21 days of age, and the severe harmful effects of AFB_1_ on performance only occurred at 2000 ppb. It has been reported that an average of 950 ppb of AFB_1_ reduces the FI and BWG by 11% [34]; therefore, it is not necessarily unusual to observe the minimal effects of feeding 500 ppb of AFB_1_ on performance.

In the present study, AFB_1_ decreased the relative weights of breast meat and carcasses. Aflatoxin prevents essential functions such as protein and nucleic acid synthesis [40]. Disturbed amino acid utilization and impaired protein synthesis may explain the lower breast meat in this study. Furthermore, the MP treatment decreased breast meat yield; the reason for this is not readily apparent. Supplemental L-Thr increased breast meat yield significantly, which is almost similar to Ahmed et al. (2020) [31], who obtained more breast meat yield by the inclusion of L-Thr in diet (110% and 120% of the NRC, 1994 requirements) compared to the control group, but observed no significant difference between 110% and 120%. The higher breast meat yield produced by L-Thr may relate to an interaction between L-Thr and Lys, which increases the utilization of Lys for muscle development [31]. According to our findings, an average ratio of digestible L-Thr to Lys of (0.79) resulted in higher breast meat yield compared to the basal diet ratio of (0.63), which is almost equal to the Cobb 500 recommendation of (0.66). When it comes to amino acids, the most important thing is balance, rather than absolute amounts. Kidd et al. (1997) [41] showed an interaction between Thr and Lys to increase breast fillet yields (Thr: Lys ratio of approximately 70%), somewhat similar to results obtained with the 79% average in this study; therefore, the breast meat yield may increase in the range of the 70 to 79% ratio of Thr to Lys.

Additionally, the best breast meat yield and carcass percentahe were observed in T_7_ containing L-Thr and MP. The 2-way interaction effect between L-Thr and MP was significant, and it increased breast meat yield. Our results represent that the combination of L-Thr and MP in the diet is helpful to increase the breast meat yield.

The serum levels of glucose and urea were affected by AFB_1_, and an impaired glucose utilization may explain this effect. Our result is almost different from the other reports [3,42,43], which observed no changes in glucose levels with 500, 2000, and 800 ppb of AFB_1_, respectively. During the first 8 weeks, a considerable amount of urea can be synthesized by chickens that will be metabolized to uric acid production by the residual embryonic hepatic arginase, which will be decreased as birds grow [44]. Aravind et al. (2003) [44] reported a lower blood urea nitrogen in birds fed a naturally contaminated diet (aflatoxin 168 ppb, ochratoxin 8.4 ppb, zearalenone 54 ppb, and T-2 toxin 32 ppb) at 21 and 35 days of age; and concluded that an altered functional status of the liver occurred. The concentration of cholesterol, HDL, and LDL was affected by 2-way interaction effects, which means that the effect of the one experimental factor depends on the level of the other experimental factor. It has been reported that AFB_1_ restrains cholesterol biosynthesis due to liver problems and impaired lipid transport [45,46,47]. In a 2-way interaction, cholesterol concentration was raised by MP compared to AFB_1_ alone, which suggests a positive effect of MP on cholesterol under the aflatoxicosis challenge.

The negative impact of AFB_1_ on the ALT concentration was significant as a 2-way interaction; some hepatic stress may explain this effect. The positive effect of MP on the concentration of LDH in a 2-way interaction with AFB_1_ demonstrates that the inclusion of MP may be helpful for birds under a low-level aflatoxicosis. The serum enzymatic activity of AST increased with higher L-Thr, which refers to increased amino acid metabolism. However, Kolbadinejad and Rezaeipour (2020) [48] did not observe any effects of 105, 110, and 115% of L-Thr on the concentration of the AST of Ross male broiler chickens at day 35.

Similarly, Sigolo et al. (2017) [49] represented the fact that AST was not affected by the increasing levels of L-Thr above the Ross recommendation (110, 120, and 130%) at day 42. Nevertheless, other researchers observed higher levels of AST, due to the metabolism of excess amino acid, imbalanced L-Thr, or higher dietary branched-chain amino acids [33]. Other than AST, ALP was increased in the present study. Aflatoxin B_1_ increased the concentration of ALP; this agrees with the previous reports [3,19], which indicated higher levels of ALP in birds fed contaminated diets with 500 ppb of AFB_1_, due to altered liver function followed by hepatocyte damage.

Alkaline phosphatase is a zinc–metalloenzyme consisting of zinc and magnesium [50], synthesized by the liver, bone, and smaller amount in intestines and kidneys [51]. It has been reported that any serum activity of ALP mainly reflects the liver and bone problems [34]. Aflatoxin B_1_ decreased bone density, which suggests some changes in the utilization of cholecalciferol and bone mineralization. Bird (1978) [25] reported a significant interaction between AFB_1_ and vitamin D_3_ on the bone mineralization of white leghorn cockerels, using a regression equation which shows that each ppm of AFB_1_ increases the vitamin D_3_ requirements by 8.84 ICU/kg of diet; this indicates an interference with the conversion of vitamin D_3_ to its more active physiological derivatives. Correspondingly, Huff. (1980) [24] represented that bone ashes were decreased by dietary aflatoxin (2500 ppb and more) in Hubbard male broilers, and mentioned that aflatoxin inhibits the vitamin D_3_-mediated mineralization of bones, and contributes to bone development problems.

Furthermore, 2000 ppb of AFB_1_ decreased tibia and ash weight in Cobb male broiler chickens [52]. Overall, Bird. (1978) [25] and Huff (1980) [24] described the capacity of aflatoxin to decrease bone ash, the result of which was not observed in this study. However, bone density decreased by AFB_1_, which indicates that even a low concentration of AFB_1_ can interfere with bone development and strength.

On the other hand, tibia fresh weight and BW to bone weight improved by dietary MP, representing no harmful bone-related consequences in birds fed MP, which may be explained by the better utilization of minerals.

Aflatoxin B_1_ had no severe consequences on H, L, and H to L ratio; only numerical, minimal adverse effects were observed (*p* = 0.09; *p* = 0.07). It can be concluded that higher levels of aflatoxins may have enough potential to impair the birds’ usual status and expose them to stress. Our results are almost different from other reports [3,8,27,53]. It has been reported that the adverse effects of mycotoxins such as AFB_1_ on H and L are related to the effects on inflammatory and immune response, hematopoiesis, or changes in the formation of humoral substances such as cytokines [46,54]. The negative impacts of aflatoxins on L hinder antibody production and depress the antibody half-life [8].

However, the percentage of L was not significantly decreased by AFB_1_ in the present study; but the antibody titer against IBV was significantly decreased in birds fed AFB_1_ (T_2_) compared to T_1_ and T_8_. Moreover, AFB_1_ decreased the IBV titer significantly. These findings are almost contrary to [55], which observed no impacts of AFB_1_ on the IBV titer of Ross 308 male broiler chickens at 75 and 750 ppb over the course of 5 weeks. On the other hand, there was a strong negative correlation (r^2^ = 0.96) at day 42 between the IBV titer and AFB_1_ concentrations (0, 250, 500, 750 ppb) in Ross broilers, revealing that this effect might relate to the potential of AFB_1_ to inhibit RNA polymerase, and consequently, depression in protein synthesis and specific immunoglobulins [56]. Moreover, Jahanian et al. (2019) [8] observed a reduction for IBV titers (20 days of age) in birds fed aflatoxins (500, 2000 ppb) from 7 to 28 days of age. Aflatoxin B_1_ increases the activity of lysosomal enzymes of skeletal muscle and liver; this effect enhances antibody degradation; aflatoxin inhibits the phagocytic cells of the reticuloendothelial systems, which are involved in the processing of antigens, as well as cells of the bursa of fabricius involved in the initiation of the humoral response [56]. It has been expressed that lymphoid organs are vulnerable to mycotoxins because of lysosomes and hydrolytic enzymes activities. Furthermore, protein synthesis depression, particularly immunoglobulins A and G, might be the reason for an immunocompromised status induced by aflatoxins [8]. However, the alternations of immunoglobulins were not under investigation in this experiment, but IBV titers were decreased. The antibody titer against IBDV was not significantly affected; however, it was suggested that there might be a relation with immunosuppression, due to aflatoxins and severe IBDV outbreak [57]. No significant effects of L-Thr on H and L were observed.

Further research to reveal the effect and mechanism of different levels of L-Thr on H and L is warranted. The serum titers of IBV and IBDV were not affected by extra L-Thr, which was not expected, due to the more utilized L-Thr as an important component of immunoglobulins. Our results are almost different from Ahmed et al. (2020) [31], but the interaction effects of L-Thr and MP resulted in a higher IBV titer, which can be interpreted as a synergistic effect. Moreover, supplemental L-Thr and MP treatment (T_8_) showed a higher IBV titer than T_2_, T_4_, and T_6_, indicating the efficacy of L-Thr and MP under an aflatoxicosis challenge. No significant effects of MP on H and L were observed, which agrees with other reports [36,58]. Despite a positive non-significant effect of Mycofix (2.5 g/kg of diet) on H and L, better physiological stress responses can be concluded [58]. Higher IBV titer in a 2-way interaction between MP and AFB_1_ demonstrates the ability of MP to counter the consequences of aflatoxicosis.

It has been reported that increasing levels of L-Thr improved the antioxidant capacity [33,59] by the best effects at 125% of the NRC; 1994 [32] recommended amounts. In the present study, the concentrations of SOD and CAT were not affected by the treatments, but a markable positive change in the GPX level was observed.

As the main effect, supplemental L-Thr significantly increased the concentration of GPX by almost 17.49%, indicating an enhanced antioxidant capacity.

Serum antioxidant capacity was not altered by the low level of AFB_1_, which almost agrees with Li et al. (2014) [18].

Some reports indicated improved gut health by dietary L-Thr more than recommended requirements [31,33,35], but intestinal morphometry was not affected by the dietary L-Thr in the present study. Despite a series of reports [19,21,22,23,60], AFB_1_ had no harmful effects on the intestinal morphometry, which is almost similar to Chen et al. (2016) [61]. However, due to the different intestinal sections, length of exposure, or species, the consequences of AFB_1_ on intestinal morphology are not wholly conclusive [23], and further research should be carried out to extend these findings.

No harmful effects of AFB_1_ on cecal microflora were observed in the present study. Galarza-Seeber et al. (2016) [27] reported inconsistent effects of AFB_1_ on cecal microflora by more than 500 ppb. Moreover, Liu et al. (2018) [4,7] indicated that 40 ppb of AFB_1_ significantly increased the *Clostridium perfringens (C. perfringens)*, *E. coli*, and Gram-negative bacteria of ileal digesta in Arbor Acres broiler chickens at 21 and 42 days of age, respectively. Aflatoxin B_1_ can affect intestinal function by mechanisms such as toxin secretion, toxin cytotoxicity, and genotoxicity in broilers [4]. On the other hand, Liu et al. (2018) [6] did not observe any significant effects of AFB_1_ (40 ppb) on the ileal populations of *Lactobacilli*, *Bifidobacteria*, *C. perfringens*, and *E. coli* of Cobb male broilers compared to control at day 21. However, the microbiota of ileal digesta were not under investigation in the present study. The cecal population was not affected by AFB_1_, but extra L-Thr numerically reduced the population of *E. coli* compared to the AFB_1_ treatment. Finally, differences among species, sex, age, diets, management, length of exposure to aflatoxins, and *Aspergillus* species, or methods used in studies, induce different responses to aflatoxicosis (similar or identical concentrations of aflatoxins).

## 4. Conclusions

Aflatoxin B_1_ did not affect the performance in this study. However, the breast meat yield and carcass percentage, glucose and urea metabolism, serum ALP, IBV titer, and bone density were negatively affected by AFB_1_. L-Threonine and MP treatment improved the breast meat yield and carcass percentage. Supplemental L-Thr and MP were helpful to improve the impaired immune response of broilers exposed to AFB_1_. Dietary L-Thr was useful for raising serum antioxidant capacity. Mycofix Plus improved some of the tibia characteristics, regardless of AFB_1_ concentration. The supplemental MP corrected the serum cholesterol and LDH levels in a 2-way interaction with AFB_1_.

An industry-relevant aflatoxicosis had almost minimal consequences in Cobb 500 broiler chickens over the course of 5 weeks. However, the negative effect of AFB_1_ on breast meat yield and carcass percentage is a significant concern, and further investigations are warranted. The authors suggest that severe harmful effects of AFB_1_ up to 500 ppb can be observed in the long term as chronic aflatoxicosis.

## 5. Materials and Methods

### 5.1. Experimental Design, Birds and Diets

A 2 × 2 × 2 factorial arrangement in a completely randomized design with 8 replicates was conducted to evaluate the efficacy of L-Thr and MP under a low-level aflatoxicosis for 5 weeks. A total of 640 1-day-old Cobb 500 male and female broiler chickens were allocated to 64 experimental units (1 m × 1.2 m) with 10 birds per unit. Feed and water consumption were “ad libitum,” and temperature, humidity, and lighting programs were performed according to the Cobb 500 management guide. Two corn–soybean meal-based basal diets were carefully formulated to meet the desired requirements to provide 100 and 125 percent of L-Thr for each stage of the production (Table 14). Treatments were as follows: T_1_ basal diet (L-Thr, 100), T_2_ (T_1_ + AFB_1_), T_3_ (T_1_ + MP), T_4_ (T_1_ + AFB_1_ + MP), T_5_ basal diet (L-Thr, 125), T_6_ (T_5_ + AFB_1_), T_7_ (T_5_ + MP), and T_8_ (T_5_ + AFB_1_ + MP). Corn grain was analyzed for AMEn and digestible amino acids, and soybean meal was analyzed for digestible amino acids by near-infrared spectroscopy (FOSS NIRS-DS2500, 91744463, Denmark).

The nutrient compositions of basal diets were analyzed for crude protein, calcium, and total phosphorous by AOAC methods [62]. All procedures involving animals were approved by the Department of Animal Science and the Research Council of Islamic Azad University, Shabestar, Iran (code: 162305888; date of approval: 12 June 2018).

### 5.2. Aflatoxin Production

*Aspergillus parasiticus* (PTCC-5286) was purchased from the Iranian Research Organization for Science and Technology (IROST) to produce aflatoxin by fermentation. The protocols [63] were performed in the Department of Poultry Science, Tarbiat Modares University, Tehran, Iran. Briefly, 100 of 1000 mL-inoculated Erlenmeyer flasks (100 g of white rice/flask; 100 mL water/flask) containing 200 mL (2 mL/flask) of *A. parasiticus* suspension (6.5 × 106 spores/mL) were incubated for 7 days at 28 °C for the fermentation process. The rice grains were autoclaved to kill the spores, and then were dried and grounded [63]. The concentration of aflatoxins was measured by HPLC at the end of the experiment. Aflatoxin assays were conducted based on the Institute of Standards and Industrial Research of Iran (ISIRI 6872), according to Mazaheri. (2009) [64]. In summary, 50 g of sample was extracted with 200 mL of methanol–water (80:20), then diluted with water and filtered through a glass microfiber filter. AflaTest WB immunoaffinity columns (IACs) were used for purification. Ten ml of phosphate buffer saline and 75 mL of the filtrate were passed through the IAC at a ca. 1 drop per second flow rate. For elution, 0.5 and 1.0 mL of methanol were passed through the column by gravity, and collected as the first and second portions, respectively. After dilution with water, reverse-phase HPLC (C_18_) and fluorescence detector with post-column derivatization (Kobra Cell) involving bromination were used to analyze aflatoxin via injection of 100 µL into HPLC. The excitation wavelength of 365 nm and emission wavelength of 435 nm were used for detection.

Finally, calculated amounts of moldy rice powder were carefully incorporated into the basal diets to reach the desired concentration in each production period (AFB_1_, 500 ppb) using a horizontal mixer. The analyzed and calculated concentrations of aflatoxins are presented in Table 15.

### 5.3. Performance, Carcass Traits and Blood Biochemical Parameters

Cumulative FI, BWG, and FCR were recorded per experimental unit and were calculated per bird for each period of production. European Production Efficiency Factor, and EBI, respectively, were calculated according to [65,66] by using the following formulas:

(1) EPEF=(Survival Rate×Final Body Weight (BW)) ÷ (Age×FCR)×100

(2) EBI=(Daily BWG×Survival Rate) ÷ (FCR×10) 

where *Survival Rate* = 100-mortality %; *Final BW* = average *BW* in kilogram at the end of the period; *Age* = market age or age at the end of the period; *FCR* = feed conversion ratio. At 35 days of age, one bird was selected per experimental unit and euthanized by cervical dislocation, then dissected to record the carcass traits, such as relative weights of breast, drumsticks, WBNT, carcass, liver, spleen, kidneys, bursa of fabricius, pancreas, heart, gizzard, and abdominal fat, after collecting blood samples by the puncture of the right-wing vein using injection syringes and sample tubes. Blood samples were centrifuged (Centrifuge, Hermle Z320, Germany) for 12 min at 3200 RPM (1500× *g*) to obtain serums, and then stored at −20 °C until the analysis. After thawing, blood serums were assessed for glucose, cholesterol, triglycerides, uric acid, urea, total protein, albumin, AST, ALT, ALP, and LDH using commercial kits (Pars Azmun Company, Karaj, Iran) by an auto-analyzer (Technicon RA-XT, Oakland, CA, USA). The serum globulin concentration was calculated by subtracting the albumin from the total protein, then the albumin to globulin ratio was calculated. High-density lipoprotein was measured using the same kits by spectrophotometry (Spectrophotometer, Jenway 6300, UK). Very low-density lipoprotein and LDL were calculated by Friedwald’s equations [67]:

(3) VLDL=triglycerides ÷ 5

(4) LDL=total cholesterol−(HDL+VLDL)

### 5.4. Differential Diagnosis of H and L

At the end of the rearing period, 64 blood samples were collected by puncturing the right-wing vein using injection syringes and EDTA tubes. Blood smears were fixed with methanol, and after drying, stained with Wrights–Giemsa stain (Water, 9 mL + Stain, 1 mL). The samples were counted for about 60 leukocytes [68] under 1000× total magnification with an optical microscope (Olympus CHK, Taiwan) and immersion oil. The H to L ratio was calculated by dividing the percentage of H to L.

### 5.5. Antibody Titer and Antioxidant Capacity

All birds were vaccinated against IBDV (16 days of age) and IBV (20 days of age) in drinking water. Blood samples were obtained at 30 and 35 days of age, respectively. Samples were centrifuged (Centrifuge, Hermle Z320, Germany) for 12 min at 3200 RPM (1500× *g*) to obtain serums, then were stored at −20 °C until the analysis. After thawing, blood serums were assessed for IBDV and IBV by a microplate reader (MPR4 Plus, Hiperion, Germany) with indirect ELISA Diagnostic Kits (IBDV, Lot-680-012; IBV, Lot-679-018, ID.vet, France). Superoxide dismutase, GPX, and CAT were measured using colorimetric assay kits (Cat-ZB-SOD-96A Lot-ZB-A5191121; Cat-ZB-GPX-A96 Lot-ZB-A7191210; Cat-ZB-CAT-96A Lot-ZB-A4191127, ZellBio GmbH, Lonsee, Germany) according to the protocols of the manufacturers.

### 5.6. Meat Quality

All of the protocols were based on Castellini et al. (2002) [69]. About 1 g of raw breast meat was homogenized for 30 s in 10 mL of 5 M iodoacetate; then, pH was measured with a digital pHmeter (Shimaz Company, Tehran, Iran). For estimation of WHC, 1 g of raw breast meats was centrifuged on tissue paper for 4 min at (1500× *g*), and dried overnight at 70 °C (Elektro-Helios, Stockholm, Sweden). Water holding capacity was calculated by the following formula [69]:

(5) (weight after centrifugation−weight after drying) ÷ initial weight×100

About 20 g of breast meats were placed in aluminum pans; then, they were cooked for 15 min in a pre-heated oven (200 °C) to reach an internal temperature of 75 °C (the most reported temperature). Cooked samples were cooled at 15 °C for 30 min and were weighed. The differences between the initial and the final weights were calculated for the estimation of cook loss. Cook loss was expressed as a percentage of the initial weight. About 15 g of raw breast and drumstick meats were stored at 4 °C for 10 days to measure lipid oxidation. Ten grams of breast and drumstick were separately homogenized with 95.7 mL of distilled water and 2.5 mL of 4 N hydrochloric acid for 2 min. The mixtures were distilled to reach 50 mL; then, 5 mL of distillate and 5 mL of thiobarbituric acid-reactive reagent (15% trichloroacetic acid and 0.375% thiobarbituric acid) were heated in a water bath for 35 min. The mixtures were then cooled under tap water for 10 min, and the absorbance was read at 538 nm (Spectrophotometer, Jenway 6300, UK) against an appropriate blank sample to obtain thiobarbituric acid-reactive substances values by multiplying optical density by 7.843. The final products were expressed as mg MDA per kg of meat [69].

### 5.7. Tibia Characteristics

Left tibia samples were carefully defleshed and cleaned of soft tissues and fibula at 35 days of age and weighed. Length and thickness (mid-point) measured by an electronic caliper (Insize 1112-150, Suzhou, China); then, the Robusticity Index and bone density were calculated according to Hafeez et al. (2014) [70] by the following formulae:

(6) Robusticity index=bone length ÷ cube root of bone weight

(7) $$\begin{array}{ll} Bone\;density= & weight\;of\;bone\;in\;air\;÷\;(weight\;of\;bone\;in\;air\\ & -\;weight\;of\;bone\;in\;water)\\ & \times \;water\;density\;at\;water\;temperature \end{array}$$

Samples were wrapped in saline-soaked gauze, then stored at −20 °C [71] until the next step. After equilibrating to room temperature and drying for 3 h in an oven (Elektro-Helios, Stockholm, Sweden) at 100 °C, tibias were defatted by immersion in petroleum ether for 48 h [72], and dried again for 12 h at 110 °C [73], and weighed before burning in a muffle furnace (Thermo-Lab, Hakim Azma Tajhiz, Tehran, Iran) at 600 °C for 6 h to obtain ashes [70].

### 5.8. Intestinal Morphometry and Cecal Microflora

At 35 days of age, 0.5 cm of distal jejunum was cut, rinsed with tap water, fixed in 10% neutral buffered formalin, dehydrated automatically by a tissue processor, and embedded in paraffin, sectioned (5-µm thick), set on a glass slide stained with Alcian Blue; then, examined by light microscopy (HD Lite Camera and TCapture V 4.3 Software, Tucsen, Fuzhou, China) for morphometric analysis. Villus height was measured from the tip of the villus to the top of the lamina propria, VW was measured at the base area of each villus, and CD was measured from the base of the invagination between the villus up to the region of transition between the crypt and villus [74]. Muscularis thickness was also investigated, and overall, 80 villi were studied per treatment. Villus surface area [75], and apparent absorptive surface area [21], respectively, were calculated using these formulae:

(8) (2π) (VW/2) (VH)

(9) 3.1×VW+3.2×VH)×1 – (2×VH)

After dissection, one gram of cecal contents dissolved in 9 mL of cold-sterile normal physiological saline (Sterile Water, 1 L + NaCl, 9 g); and homogenized using falcon tubes and a vortex mixer. Each sample was serially diluted 10-fold until 10^−6^ with the normal saline (0.9% NaCl). Final diluted samples (100 µL) were inoculated by mechanical pipette (TopPette, Dragon Lab, China) into the De Man, Rogosa and Sharpe agar (MRS), Eosin Methylene Blue (EMB), and Plate Count Agar (PCA) (Ibresco, Iran) for counting *Lactobacilli*, *E. coli*, and *TAC*, respectively. Samples were incubated (Incubator SHIH 55, Shimaz Company, Tehran, Iran) for 24 h with 37 °C, and the counted colonies (Colony Counter Sana SL-902, Shimaz Company, Iran) were multiplied by 10^6^, and then expressed as the log_10_ of (CFU) g^−1^.

### 5.9. Statistical Analysis

Experimental data and residuals were checked for normality using the Kolmogorov–Smirnov test. Data were analyzed as a 3-way ANOVA model using a 2-level factorial arrangement in a completely randomized design by the general linear models procedure of SAS 9.4 (SAS Institute, Cary, NC), according to the following formula:

(10) xiklm=µ +αk+βl+ ym+(αβ)kl+(αy)km+(βy)lm+(αβy)klm+ɛiklm

where *xiklm* is the value of each observation; µ is the mean of the dependent variables; *αk*, *βl*, *ym* are the independent variables; *(αβ)kl*, *(αy)km*, *(βy)lm*, *(αβy)klm* are the interaction effects of independent variables; and *ɛiklm* is the experimental error. In the presence of main and interaction effects (*p* < 0.05), all means were compared using Duncan’s multiple range test, with a significance level of 0.05. A one-way ANOVA model was used to compare the treatments. Data tables for 2-way interactions are contained within the Supplementary Material.

## Figures and Tables

**Table 1:** Effect of L-Threonine and Mycofix Plus (MP) on performance of broilers exposed to Aflatoxin B_1_, Cobb 500.

				Starter, 1 to 8 Days			Grower, 9 to 18 Days		
Treatments	L-Threonine, % of Requirements	MP, g/kg	Aflatoxin B_1_, 500 µg/kg	FI ^1^, g	BWG ^2^, g	FCR ^3^	FI, g	BWG, g	FCR
T_1_	100	0	−	174.1	140.5	1.24	544.4 ^b^	331.2	1.64
T_2_	100	0	+	176.2	140.1	1.26	543.4 ^b^	309.6	1.77
T_3_	100	1	−	176.2	138.9	1.27	542.7 ^b^	316.4	1.72
T_4_	100	1	+	178.0	143.2	1.25	546.4 ^a,b^	316.5	1.73
T_5_	125	0	−	178.3	140.2	1.28	543.7 ^b^	306.7	1.78
T_6_	125	0	+	175.7	139.8	1.26	545.3 ^a,b^	303.1	1.81
T_7_	125	1	−	175.1	141.6	1.24	548.9 ^a^	324.8	1.71
T_8_	125	1	+	177.8	139.1	1.28	547.3 ^a,b^	305.8	1.81
Pooled SEM				2.25	3.52	0.02	1.43	9.23	0.05
Main Effects			Levels	Means ^4^					
L-Threonine			100	176.1	140.7	1.25	544.2 ^b^	318.4	1.72
			125	176.7	140.2	1.26	546.3 ^a^	310.1	1.78
Mycofix Plus			0	176.1	140.1	1.26	544.2 ^b^	312.7	1.75
			1	176.8	140.7	1.26	546.4 ^a^	315.9	1.74
Aflatoxin B_1_			−	175.9	140.3	1.26	544.9	319.8	1.71
			+	176.9	140.5	1.26	545.6	308.8	1.78
Main Effects and Interaction Effects				*p*-values					
L-Threonine				NS	NS	NS	*	NS	NS
Mycofix Plus				NS	NS	NS	*	NS	NS
Aflatoxin B_1_				NS	NS	NS	NS	NS	NS
L-Threonine × Mycofix Plus				NS	NS	NS	NS	NS	NS
L-Threonine × Aflatoxin B_1_				NS	NS	NS	NS	NS	NS
Mycofix Plus × Aflatoxin B_1_				NS	NS	NS	NS	NS	NS
L-Threonine × Mycofix Plus × Aflatoxin B_1_				NS	NS	NS	NS	NS	NS

^a,b^ Means within a column with differing superscripts are significantly different at * *p* < 0.05; NS, *p* ≥ 0.05. ^1^ Feed Intake. ^2^ Body Weight Gain. ^3^ Feed Conversion Ratio. ^4^ Means represent 32 pens of chickens with 10 birds per pen (*n* = 32/group).

**Table 2:** Effect of L-Threonine and Mycofix Plus (MP) on performance of broilers exposed to Aflatoxin B_1_, Cobb 500.

				Finisher 1, 19 to 28 Days			Finisher 2, 29 to 35 Days		
Treatmenst	L-Threonine, % of Requirements	MP, g/kg	Aflatoxin B_1_, 500 µg/kg	FI ^1^, g	BWG ^2^, g	FCR ^3^	FI, g	BWG, g	FCR
T_1_	100	0	−	1055.7	656.7	1.61	951.1	499.1	1.92
T_2_	100	0	+	1054.9	659.4	1.60	962.3	500.0	1.94
T_3_	100	1	−	1060.9	667.6	1.59	951.8	468.5	2.05
T_4_	100	1	+	1011.4	653.3	1.56	946.2	498.6	1.91
T_5_	125	0	−	1072.6	689.2	1.56	943.9	517.8	1.85
T_6_	125	0	+	1072.9	684.4	1.57	944.9	488.6	1.95
T_7_	125	1	−	1067.6	661.4	1.61	953.0	512.5	1.87
T_8_	125	1	+	1066.5	663.4	1.61	955.9	521.9	1.84
Pooled SEM				20.24	13.60	0.03	12.43	17.45	0.06
Main Effects			Levels	Means ^4^					
L-Threonine			100	1045.7	659.2	1.59	952.8	491.5	1.95
			125	1069.9	674.6	1.59	949.4	510.2	1.88
Mycofix Plus			0	1064.0	672.4	1.58	950.5	501.4	1.91
			1	1051.6	661.4	1.59	951.7	500.4	1.92
Aflatoxin B_1_			−	1064.2	668.7	1.59	949.9	499.5	1.92
			+	1051.4	665.1	1.58	952.3	502.3	1.91
Main Effects and Interaction Effects				*p*-values					
L-Threonine				NS	NS	NS	NS	NS	NS
Mycofix Plus				NS	NS	NS	NS	NS	NS
Aflatoxin B_1_				NS	NS	NS	NS	NS	NS
L-Threonine × Mycofix Plus				NS	NS	NS	NS	NS	NS
L-Threonine × Aflatoxin B_1_				NS	NS	NS	NS	NS	NS
Mycofix Plus × Aflatoxin B_1_				NS	NS	NS	NS	NS	NS
L-Threonine × Mycofix Plus × Aflatoxin B_1_				NS	NS	NS	NS	NS	NS

^1^ Feed Intake. ^2^ Body Weight Gain. ^3^ Feed Conversion Ratio. ^4^ Means represent 32 pens of chickens with 10 birds per pen. (*n* = 32/group). NS, *p* ≥ 0.05.

**Table 3:** Effect of L-Threonine and Mycofix Plus (MP) on performance of broilers exposed to Aflatoxin B_1_, Cobb 500.

				Total, 1 to 35 Days				
Treatments	L-Threonine, % of Requirements	MP, g/kg	Aflatoxin B_1_, 500 µg/kg	FI ^1^, g	BWG ^2^, g	FCR ^3^	EPEF ^4^	EBI ^5^
T_1_	100	0	−	2725.2	1627.4	1.68	269.2	261.5
T_2_	100	0	+	2736.7	1609.1	1.71	265.5	257.9
T_3_	100	1	−	2731.6	1591.4	1.72	264.2	256.4
T_4_	100	1	+	2682.0	1611.6	1.67	278.7	270.7
T_5_	125	0	−	2738.5	1653.9	1.66	277.1	269.3
T_6_	125	0	+	2738.8	1615.9	1.70	264.5	257.0
T_7_	125	1	−	2744.6	1640.3	1.68	274.5	266.7
T_8_	125	1	+	2747.5	1630.2	1.69	269.6	261.8
Pooled SEM				25.87	31.16	0.03	14.29	13.98
Main Effects			Levels	Means ^6^				
L-Threonine			100	2718.9	1609.9	1.69	269.4	261.6
			125	2742.3	1635.1	1.68	271.4	263.7
Mycofix Plus			0	2734.8	1626.6	1.68	269.1	261.4
			1	2726.4	1618.4	1.69	271.7	263.9
Aflatoxin B_1_			−	2735.0	1628.3	1.68	271.2	263.5
			+	2726.2	1616.7	1.69	269.6	261.8
Main Effects and Interaction Effects				*p*-values				
L-Threonine				NS	NS	NS	NS	NS
Mycofix Plus				NS	NS	NS	NS	NS
Aflatoxin B_1_				NS	NS	NS	NS	NS
L-Threonine × Mycofix Plus				NS	NS	NS	NS	NS
L-Threonine × Aflatoxin B_1_				NS	NS	NS	NS	NS
Mycofix Plus × Aflatoxin B_1_				NS	NS	NS	NS	NS
L-Threonine × Mycofix Plus × Aflatoxin B_1_				NS	NS	NS	NS	NS

^1^ Feed Intake. ^2^ Body Weight Gain. ^3^ Feed Conversion Ratio. ^4^ European Production Efficiency Factor. ^5^ European Broiler Index. ^6^ Means represent 32 pens of chickens with 10 birds per pen (*n* = 32/group). NS, *p* ≥ 0.05.

**Table 4:** Effect of L-Threonine and Mycofix Plus (MP) on carcass traits of broilers exposed to Aflatoxin B_1_ at day 35, Cobb 500.

				Breast	Drumsticks	WBNT ^1^	Carcass	Liver	Spleen
Treatmenst	L-Threonine, % of Requirements	MP, g/kg	Aflatoxin B_1_, 500 µg/kg	Relative Weights, % of Live Body Weight					
T_1_	100	0	−	22.75 ^a,b^	20.34	18.53	61.61 ^a,b^	2.68	0.09
T_2_	100	0	+	19.89 ^c^	19.88	18.79	58.55 ^c^	2.77	0.10
T_3_	100	1	−	20.39 ^c^	19.99	18.68	59.06 ^c^	2.89	0.10
T_4_	100	1	+	19.73 ^c^	19.97	19.27	58.97 ^c^	2.67	0.11
T_5_	125	0	−	21.14 ^b,c^	19.94	18.60	59.68 ^b,c^	2.62	0.09
T_6_	125	0	+	21.42 ^b,c^	19.29	18.41	59.12 ^c^	2.62	0.09
T_7_	125	1	−	23.32 ^a^	20.13	18.94	62.38 ^a^	2.53	0.11
T_8_	125	1	+	20.45 ^c^	19.28	18.57	58.30 ^c^	2.71	0.09
Pooled SEM				0.60	0.37	0.34	0.76	0.16	0.01
Main Effects			Levels	Means ^2^					
L-Threonine			100	20.69 ^b^	20.04	18.82	59.55	2.75	0.10
			125	21.59 ^a^	19.66	18.63	59.87	2.62	0.09
Mycofix Plus			0	21.30	19.86	18.58	59.74	2.67	0.09
			1	20.98	19.84	18.86	59.68	2.70	0.10
Aflatoxin B_1_			−	21.90 ^a^	20.10	18.68	60.68 ^a^	2.68	0.10
			+	20.37 ^b^	19.61	18.76	58.74 ^b^	2.69	0.10
Main Effects and Interaction Effects				*p*-values					
L-Threonine				*	NS	NS	NS	NS	NS
Mycofix Plus				NS	NS	NS	NS	NS	NS
Aflatoxin B_1_				***	NS	NS	***	NS	NS
L-Threonine × Mycofix Plus				*	NS	NS	NS	NS	NS
L-Threonine × Aflatoxin B_1_				NS	NS	NS	NS	NS	NS
Mycofix Plus × Aflatoxin B_1_				NS	NS	NS	NS	NS	NS
L-Threonine × Mycofix Plus × Aflatoxin B_1_				**	NS	NS	**	NS	NS

^a^^–c^ Means within a column with differing superscripts are significantly different at * *p* < 0.05; ** *p* < 0.01; *** *p* < 0.001. NS, *p* ≥ 0.05. ^1^ Wings, Back, Neck, Thigh. ^2^ Means represent 32 pens of chickens with 10 birds per pen (*n* = 32/group).

**Table 5:** Effect of L-Threonine and Mycofix Plus (MP) on carcass traits of broilers exposed to Aflatoxin B_1_ at day 35, Cobb 500.

				Kidneys	Bursa of Fabricius	Pancreas	Heart	Gizzard	Abdominal Fat
Treatmenst	L-Threonine, % of Requirements	MP, g/kg	Aflatoxin B_1_, 500 µg/kg	Relative Weights, % of Live Body Weight					
T_1_	100	0	−	0.44	0.15	0.24	0.55	1.74	1.37
T_2_	100	0	+	0.44	0.16	0.24	0.54	1.89	2.12
T_3_	100	1	−	0.50	0.16	0.25	0.60	1.78	2.01
T_4_	100	1	+	0.50	0.14	0.27	0.61	1.96	2.12
T_5_	125	0	−	0.53	0.15	0.25	0.57	1.73	1.93
T_6_	125	0	+	0.46	0.15	0.25	0.58	1.70	1.71
T_7_	125	1	−	0.51	0.19	0.26	0.59	1.83	1.79
T_8_	125	1	+	0.41	0.16	0.27	0.59	1.79	1.64
Pooled SEM				0.04	0.02	0.01	0.03	0.08	0.25
Main Effects			Levels	Means ^1^					
L-Threonine			100	0.47	0.15	0.25	0.57	1.84	1.90
			125	0.48	0.16	0.26	0.58	1.76	1.77
Mycofix Plus			0	0.47	0.15	0.24	0.56	1.76	1.78
			1	0.48	0.16	0.26	0.60	1.84	1.89
Aflatoxin B_1_			−	0.49	0.16	0.25	0.58	1.77	1.77
			+	0.45	0.15	0.26	0.58	1.83	1.90
Main Effects and Interaction Effects				*p*-values					
L-Threonine				NS	NS	NS	NS	NS	NS
Mycofix Plus				NS	NS	NS	NS	NS	NS
Aflatoxin B_1_				NS	NS	NS	NS	NS	NS
L-Threonine × Mycofix Plus				NS	NS	NS	NS	NS	NS
L-Threonine × Aflatoxin B_1_				NS	NS	NS	NS	NS	NS
Mycofix Plus × Aflatoxin B_1_				NS	NS	NS	NS	NS	NS
L-Threonine × Mycofix Plus × Aflatoxin B_1_				NS	NS	NS	NS	NS	NS

^1^ Means represent 32 pens of chickens with 10 birds per pen (*n* = 32/group). NS, *p* ≥ 0.05.

**Table 6:** Effect of L-Threonine and Mycofix Plus (MP) on blood biochemical parameters of broilers exposed to Aflatoxin B_1_ at day 35, Cobb 500.

				Glucose	Cholesterol	Triglycerides	HDL ^1^	LDL ^2^	VLDL ^3^
Treatmenst	L-Threonine, % of Requirements	MP, g/kg	Aflatoxin B_1_, 500 µg/kg	mg/dL	mg/dL	mg/dL	mg/dL	mg/dL	mg/dL
T_1_	100	0	−	208.38	130.21	96.38	83.50	27.44	19.28
T_2_	100	0	+	247.88	117.05	96.85	76.38	21.31	19.37
T_3_	100	1	−	218.50	123.20	94.81	76.75	27.49	18.96
T_4_	100	1	+	226.38	134.35	87.24	76.63	40.28	17.45
T_5_	125	0	−	224.63	133.60	91.04	77.13	38.27	18.21
T_6_	125	0	+	234.38	128.79	111.83	72.25	34.17	22.37
T_7_	125	1	−	204.50	126.43	99.38	77.25	29.30	19.88
T_8_	125	1	+	225.38	132.24	106.16	81.13	29.88	21.23
Pooled SEM				12.22	4.77	10.49	2.67	4.86	2.10
Main Effects			Levels	Means ^4^					
L-Threonine			100	225.28	126.20	93.82	78.31	29.13	18.76
			125	222.22	130.26	102.10	76.94	32.91	20.42
Mycofix Plus			0	228.81	127.41	99.02	77.31	30.30	19.80
			1	218.69	129.05	96.90	77.94	31.74	19.38
Aflatoxin B_1_			−	214.00 ^b^	128.36	95.40	78.66	30.62	19.08
			+	233.50 ^a^	128.11	100.52	76.59	31.41	20.10
Main Effects and Interaction Effects				*p*-values					
L-Threonine				NS	NS	NS	NS	NS	NS
Mycofix Plus				NS	NS	NS	NS	NS	NS
Aflatoxin B_1_				*	NS	NS	NS	NS	NS
L-Threonine × Mycofix Plus				NS	NS	NS	NS	*	NS
L-Threonine × Aflatoxin B_1_				NS	NS	NS	NS	NS	NS
Mycofix Plus × Aflatoxin B_1_				NS	*	NS	*	NS	NS
L-Threonine × Mycofix Plus × Aflatoxin B_1_				NS	NS	NS	NS	NS	NS

^a,b^ Means within a column with differing superscripts are significantly different at * *p* < 0.05. NS, *p* ≥ 0.05. ^1^ High-Density Lipoprotein. ^2^ Low-Density Lipoprotein. ^3^ Very Low-Density Lipoprotein. ^4^ Means represent 32 pens of chickens with 10 birds per pen. (*n* = 32/group).

**Table 7:** Effect of L-Threonine and Mycofix Plus (MP) on blood biochemical parameters of broilers exposed to Aflatoxin B_1_ at day 35, Cobb 500.

				Uric Acid	Urea	Total Protein	Albumin	Globulin	A/G ^1^
Treatmenst	L-Threonine, % of Requirements	MP, g/kg	Aflatoxin B_1_, 500 µg/kg	mg/dl	mg/dL	g/dL	g/dL	g/dL	
T_1_	100	0	−	3.53	2.69	3.97	1.26	2.72	0.47
T_2_	100	0	+	4.46	1.54	3.85	1.16	2.69	0.43
T_3_	100	1	−	4.30	2.41	4.17	1.18	2.99	0.41
T_4_	100	1	+	3.43	2.20	3.50	1.06	2.44	0.44
T_5_	125	0	−	4.29	1.96	4.10	1.31	2.79	0.47
T_6_	125	0	+	3.85	1.93	3.82	1.12	2.70	0.43
T_7_	125	1	−	4.50	2.39	3.77	1.17	2.61	0.45
T_8_	125	1	+	4.05	1.72	4.03	1.30	2.73	0.49
Pooled SEM				0.33	0.32	0.21	0.07	0.17	0.03
Main Effects			Levels	Means ^2^					
L-Threonine			100	3.93	2.21	3.87	1.16	2.71	0.44
			125	4.17	2.00	3.93	1.22	2.71	0.46
Mycofix Plus			0	4.03	2.03	3.93	1.21	2.72	0.45
			1	4.07	2.18	3.87	1.18	2.69	0.44
Aflatoxin B_1_			−	4.15	2.36 ^a^	4.00	1.23	2.78	0.45
			+	3.94	1.85 ^b^	3.80	1.16	2.64	0.45
Main Effects and Interaction Effects				*p*-values					
L-Threonine				NS	NS	NS	NS	NS	NS
Mycofix Plus				NS	NS	NS	NS	NS	NS
Aflatoxin B_1_				NS	*	NS	NS	NS	NS
L-Threonine × Mycofix Plus				NS	NS	NS	NS	NS	NS
L-Threonine × Aflatoxin B_1_				NS	NS	NS	NS	NS	NS
Mycofix Plus × Aflatoxin B_1_				NS	NS	NS	NS	NS	NS
L-Threonine × Mycofix Plus × Aflatoxin B_1_				NS	NS	NS	NS	NS	NS

^a,b^ Means within a column with differing superscripts are significantly different at * *p* < 0.05. NS, *p* ≥ 0.05. ^1^ Albumin to Globulin. ^2^ Means represent 32 pens of chickens with 10 birds per pen (*n* = 32/group).

**Table 8:** Effect of L-Threonine and Mycofix Plus (MP) on serum enzymatic activity of broilers exposed to Aflatoxin B_1_ at day 35, Cobb 500.

				AST ^1^	ALT ^2^	ALP ^3^	LDH ^4^
Treatments	L-Threonine, % of Requirements	MP, g/kg	Aflatoxin B_1_, 500 µg/kg	u/L	u/L	u/L	u/L
T_1_	100	0	−	192.50	4.04	1789.00	910.75
T_2_	100	0	+	180.00	5.60	2047.50	1017.75
T_3_	100	1	−	158.50	4.39	1923.50	990.00
T_4_	100	1	+	176.75	4.10	2015.50	672.75
T_5_	125	0	−	192.88	3.73	1961.25	711.25
T_6_	125	0	+	191.38	5.01	1917.25	1132.00
T_7_	125	1	−	188.38	5.31	1987.75	883.38
T_8_	125	1	+	207.38	4.16	2135.00	848.00
Pooled SEM				12.45	0.65	71.03	134.78
Main Effects			Levels	Means ^5^			
L-Threonine			100	176.94 ^b^	4.53	1943.88	897.81
			125	195.00 ^a^	4.55	2000.31	893.66
Mycofix Plus			0	189.19	4.59	1928.75	942.94
			1	182.75	4.49	2015.44	848.53
Aflatoxin B_1_			−	183.06	4.37	1915.38 ^b^	873.84
			+	188.88	4.72	2028.81 ^a^	917.63
Main Effects and Interaction Effects				*p*-values			
L-Threonine				*	NS	NS	NS
Mycofix Plus				NS	NS	NS	NS
Aflatoxin B_1_				NS	NS	*	NS
L-Threonine × Mycofix Plus				NS	NS	NS	NS
L-Threonine × Aflatoxin B_1_				NS	NS	NS	NS
Mycofix Plus × Aflatoxin B_1_				NS	*	NS	*
L-Threonine × Mycofix Plus × Aflatoxin B_1_				NS	NS	NS	NS

^a,b^ Means within a column with differing superscripts are significantly different at * *p* < 0.05. NS, *p* ≥ 0.05. ^1^ Aspartate transaminase. ^2^ Alanine aminotransferase. ^3^ Alkaline phosphatase. ^4^ Lactate dehydrogenase. ^5^ Means represent 32 pens of chickens with 10 birds per pen (*n* = 32/group).

**Table 9:** Effect of L-Threonine and Mycofix Plus (MP) on stress status and serum anti-body titer ^1^ of broilers exposed to Aflatoxin B_1_, Cobb 500.

				Heterophil	Lymphocyte	H:L ^2^	IBV ^3^	IBDV ^4^
Treatmenst	L-Threonine, % of Requirements	MP, g/kg	Aflatoxin B_1_, 500 µg/kg	%	%		log_10_	log_10_
T_1_	100	0	−	32.08	67.92	0.47	3.833 ^a^	3.675
T_2_	100	0	+	34.79	65.21	0.54	3.824 ^b,c^	3.723
T_3_	100	1	−	32.08	67.92	0.47	3.826 ^a,b^	3.737
T_4_	100	1	+	32.50	67.50	0.48	3.821 ^b,c^	3.667
T_5_	125	0	−	32.29	67.71	0.48	3.828 ^a,b^	3.669
T_6_	125	0	+	32.50	67.50	0.48	3.818 ^c^	3.639
T_7_	125	1	−	32.08	67.92	0.48	3.826 ^a,b^	3.657
T_8_	125	1	+	35.21	64.79	0.55	3.833 ^a^	3.675
Pooled SEM				1.30	1.30	0.03	0.003	0.06
Main Effects			Levels	Means ^5^				
L-Threonine			100	32.86	67.14	0.49	3.826	3.701
			125	33.02	66.98	0.50	3.826	3.660
Mycofix Plus			0	32.92	67.08	0.49	3.826	3.676
			1	32.97	67.03	0.50	3.827	3.684
Aflatoxin B_1_			−	32.14	67.86	0.48	3.828 ^a^	3.685
			+	33.75	66.25	0.51	3.824 ^b^	3.676
Main Effects and Interaction Effects				*p*-values				
L-Threonine				NS	NS	NS	NS	NS
Mycofix Plus				NS	NS	NS	NS	NS
Aflatoxin B_1_				NS	NS	NS	*	NS
L-Threonine × Mycofix Plus				NS	NS	NS	**	NS
L-Threonine × Aflatoxin B_1_				NS	NS	NS	NS	NS
Mycofix Plus × Aflatoxin B_1_				NS	NS	NS	**	NS
L-Threonine × Mycofix Plus × Aflatoxin B_1_				NS	NS	NS	NS	NS

^a,b^ Means within a column with differing superscripts are significantly different at * *p* < 0.05; ** *p* < 0.01. NS, *p* ≥ 0.05. ^1^ Blood samples for measuring IBDV titers were collected at day 30. ^2^ Heterophil to Lymphocyte. ^3^ Infectious Bronchitis Virus. ^4^ Infectious Bursal Disease Virus. ^5^ Means represent 32 pens of chickens with 10 birds per pen (*n* = 32/group).

**Table 10:** Effect of L-Threonine and Mycofix Plus (MP) on serum antioxidant capacity of broilers exposed to Aflatoxin B_1_, Cobb 500.

				SOD ^1^	GPX ^2^	CAT ^3^
Treatments	L-Threonine, % of Requirements	MP, g/kg	Aflatoxin B_1_, 500 µg/kg	u/mL	u/mL	u/mL
T_1_	100	0	−	14.89	564.29	7.91
T_2_	100	0	+	14.37	518.98	6.01
T_3_	100	1	−	13.16	648.57	5.68
T_4_	100	1	+	14.23	651.32	6.87
T_5_	125	0	−	16.05	649.48	6.21
T_6_	125	0	+	13.71	686.27	5.62
T_7_	125	1	−	15.37	735.98	7.80
T_8_	125	1	+	14.43	728.32	7.44
Pooled SEM				1.25	69.28	1.37
Main Effects			Levels	Means ^4^		
L-Threonine			100	14.16	595.79 ^b^	6.62
			125	14.89	700.01 ^a^	6.77
Mycofix Plus			0	14.75	604.75	6.44
			1	14.30	691.05	6.95
Aflatoxin B_1_			+	14.87	649.58	6.90
			−	14.18	646.22	6.48
Main Effects and Interaction Effects				*p*-values		
L-Threonine				NS	*	NS
Mycofix Plus				NS	NS	NS
Aflatoxin B_1_				NS	NS	NS
L-Threonine × Mycofix Plus				NS	NS	NS
L-Threonine × Aflatoxin B_1_				NS	NS	NS
Mycofix Plus × Aflatoxin B_1_				NS	NS	NS
L-Threonine × Mycofix Plus × Aflatoxin B_1_				NS	NS	NS

^a,b^ Means within a column with differing superscripts are significantly different at * *p* < 0.05. NS, *p* ≥ 0.05. ^1^ Superoxide dismutase. ^2^ Glutathione peroxidase. ^3^ Catalase. ^4^ Means represent 32 pens of chickens with 10 birds per pen (*n* = 32/group).

**Table 11:** Effect of L-Threonine and Mycofix Plus (MP) on tibia characteristics of broilers exposed to Aflatoxin B_1_ at day 35, Cobb 500.

				Fresh Weight ^1^	Fat Free Dry Weight ^1^	Ash ^2^	BW ^3^: Bone Weight
Treatments	L-Threonine, % of Requiremens	MP, g/kg	Aflatoxin B_1_, 500 µg/kg	%	%	%	
T_1_	100	0	−	0.49	0.20	51.72	205.99
T_2_	100	0	+	0.48	0.20	50.33	210.02
T_3_	100	1	−	0.53	0.21	50.18	189.64
T_4_	100	1	+	0.52	0.21	51.20	191.84
T_5_	125	0	−	0.48	0.19	50.07	208.17
T_6_	125	0	+	0.52	0.21	50.36	194.95
T_7_	125	1	−	0.50	0.21	51.87	202.71
T_8_	125	1	+	0.51	0.20	49.62	197.96
Pooled SEM				0.02	0.01	0.57	6.42
Main Effects			Levels	Means ^4^			
L-Threonine			100	0.51	0.20	50.86	199.37
			125	0.50	0.20	50.48	200.95
Mycofix Plus			0	0.49 ^b^	0.20	50.62	204.78 ^a^
			1	0.52 ^a^	0.20	50.72	195.54 ^b^
Aflatoxin B_1_			−	0.50	0.20	50.96	201.63
			+	0.51	0.20	50.38	198.69
Main Effects and Interaction Effects				*p*-values			
L-Threonine				NS	NS	NS	NS
Mycofix Plus				*	NS	NS	*
Aflatoxin B_1_				NS	NS	NS	NS
L-Threonine × Mycofix Plus				NS	NS	NS	NS
L-Threonine × Aflatoxin B_1_				NS	NS	NS	NS
Mycofix Plus × Aflatoxin B_1_				NS	NS	NS	NS
L-Threonine × Mycofix Plus × Aflatoxin B_1_				NS	NS	NS	NS

^a,b^ Means within a column with differing superscripts are significantly different at * *p* < 0.05. NS, *p* ≥ 0.05. ^1^ Percentage of Live Body Weight. ^2^ Percentage of Defatted Dry Tibia Weight. ^3^ Body Weight. ^4^ Means represent 32 pens of chickens with 10 birds per pen. (*n* = 32/group).

**Table 12:** Effect of L-Threonine and Mycofix Plus (MP) on tibia characteristics of broilers exposed to Aflatoxin B_1_ at day 35, Cobb 500.

				Length	Thickness	Robusticity Index	Density
Treatments	L-Threonine, % of Requirements	MP, g/kg	Aflatoxin B_1_, 500 µg/kg	cm	cm		g/cm^3^
T_1_	100	0	−	9.03	0.85	4.13	1.16
T_2_	100	0	+	8.88	0.78	4.16	1.15
T_3_	100	1	−	9.18	0.86	4.08	1.21
T_4_	100	1	+	8.82	0.84	4.13	1.13
T_5_	125	0	−	8.95	0.82	4.16	1.21
T_6_	125	0	+	8.89	0.86	4.13	1.16
T_7_	125	1	−	8.88	0.84	4.13	1.20
T_8_	125	1	+	8.83	0.81	4.14	1.19
Pooled SEM				0.12	0.03	0.03	0.02
Main Effects			Levels	Means ^1^			
L-Threonine			100	8.98	0.83	4.12	1.16
			125	8.89	0.83	4.14	1.19
Mycofix Plus			0	8.94	0.83	4.14	1.17
			1	8.93	0.84	4.12	1.18
Aflatoxin B_1_			−	9.01	0.84	4.12	1.20 ^a^
			+	8.86	0.82	4.14	1.16 ^b^
Main Effects and Interaction Effects				*p*-values			
L-Threonine				NS	NS	NS	NS
Mycofix Plus				NS	NS	NS	NS
Aflatoxin B_1_				NS	NS	NS	*
L-Threonine × Mycofix Plus				NS	NS	NS	NS
L-Threonine × Aflatoxin B_1_				NS	NS	NS	NS
Mycofix Plus × Aflatoxin B_1_				NS	NS	NS	NS
L-Threonine × Mycofix Plus × Aflatoxin B_1_				NS	NS	NS	NS

^a,b^ Means within a column with differing superscripts are significantly different at * *p* < 0.05. NS, *p* ≥ 0.05. ^1^ Means represent 32 pens of chickens with 10 birds per pen (*n* = 32/group).

**Table 13:** Effect of L-Threonine and Mycofix Plus (MP) on jejunal morphometry of broilers exposed to Aflatoxin B_1_ at day 35, Cobb 500.

				Villus Height	Villus Width	Crypt Depth	VH:CD ^1^	Muscular Layer	Surface Area	Apparent Absorptive Surface Area
Treatments	L-Threonine, % of Requirements	MP, g/kg	Aflatoxin B_1_, 500 µg/kg	µm	µm	µm		µm	µm^2^	µm^2^
T_1_	100	0	−	1112.89	204.14	240.75	4.99	172.66	718,299	1968.3
T_2_	100	0	+	1229.83	201.13	237.08	5.47	152.13	761,173	2099.3
T_3_	100	1	−	1182.54	209.36	240.03	5.19	168.44	795,178	2068.1
T_4_	100	1	+	1133.84	187.84	225.14	5.43	158.42	663,192	1942.9
T_5_	125	0	−	1115.22	201.79	226.37	5.25	174.40	710,441	1963.8
T_6_	125	0	+	1174.73	207.81	237.51	5.19	141.63	774,398	2053.9
T_7_	125	1	−	1244.00	188.41	220.87	5.97	161.44	724,213	2076.9
T_8_	125	1	+	1205.36	206.20	239.13	5.34	157.94	777,047	2085.7
Pooled SEM				85.13	10.54	13.55	0.41	12.52	66,123.29	107.58
Main Effects			Levels	Means ^2^						
L-Threonine			100	1164.77	200.62	235.75	5.27	162.91	734,460	2019.6
			125	1184.83	201.06	230.97	5.43	158.85	746,525	2045.1
Mycofix Plus			0	1158.16	203.72	235.43	5.22	160.20	741,078	2021.3
			1	1191.43	197.95	231.29	5.48	161.56	739,908	2043.4
Aflatoxin B_1_			−	1163.66	200.93	232.00	5.35	169.23	737,033	2019.3
			+	1185.94	200.75	234.72	5.36	152.53	743,953	2045.4
Main Effects and Interaction Effects				*p*-values						
L-Threonine				NS	NS	NS	NS	NS	NS	NS
Mycofix Plus				NS	NS	NS	NS	NS	NS	NS
Aflatoxin B_1_				NS	NS	NS	NS	NS	NS	NS
L-Threonine × Mycofix Plus				NS	NS	NS	NS	NS	NS	NS
L-Threonine × Aflatoxin B_1_				NS	NS	NS	NS	NS	NS	NS
Mycofix Plus × Aflatoxin B_1_				NS	NS	NS	NS	NS	NS	NS
L-Threonine × Mycofix Plus × Aflatoxin B_1_				NS	NS	NS	NS	NS	NS	NS

^1^ Villus Height to Crypt Depth. ^2^ Means represent 32 pens of chickens with 10 birds per pen (*n* = 32/group). NS, *p* ≥ 0.05.

**Table 14:** Composition of the diets ^1^, analyzed and calculated nutrients in different periods of the experiment, including two levels of L-Threonine (100 and 125% of the requirements, Cobb 500).

Ingredients, %	Starter, 1 to 8 Days		Grower, 9 to 18 Days		Finisher 1, 19 to 28 Days		Finisher 2, 29 to 35 Days	
	100%	125%	100%	125%	100%	125%	100%	125%
Corn grain	55.42	55.32	60.32	60.40	62.10	62.10	63.77	63.81
Soybean meal (44% CP)	35.87	35.75	31.11	30.85	28.75	28.58	26.40	26.20
Soybean oil	3.78	3.78	4.20	4.20	5.10	5.10	5.80	5.80
Calcium carbonate	1.17	1.17	0.82	0.82	0.76	0.76	0.76	0.76
Dicalcium phosphate	2.04	2.04	1.92	1.92	1.72	1.72	1.74	1.74
Sodium bicarbonate	0.17	0.17	0.18	0.18	0.18	0.18	0.18	0.18
Salt	0.29	0.29	0.29	0.29	0.29	0.29	0.29	0.29
Methionine ^2^	0.35	0.35	0.32	0.32	0.30	0.30	0.28	0.28
Lysine ^2^	0.42	0.42	0.38	0.38	0.38	0.38	0.36	0.36
L-threonine ^2^	0.09	0.31	0.06	0.24	0.02	0.19	0.02	0.18
Mineral premix ^3^	0.20	0.20	0.20	0.20	0.20	0.20	0.20	0.20
Vitamin premix ^3^	0.20	0.20	0.20	0.20	0.20	0.20	0.20	0.20
	Nutrients Composition							
AME_n_, Kcal/kg	2911	2913	2992	2995	3072	3074	3132	3134
CP, % (Analyzed)	21	21	19	19	18	18	17	17
Calcium, % (Analyzed)	0.90	0.90	0.84	0.84	0.77	0.77	0.76	0.76
Total phosphorous. % (Analyzed)	0.76	0.76	0.71	0.71	0.64	0.64	0.63	0.63
Available phosphorous, %	0.45	0.45	0.42	0.42	0.38	0.38	0.38	0.38
Digestible threonine, %	0.83	1.04	0.73	0.91	0.66	0.83	0.63	0.79
Digestible arginine, %	1.34	1.34	1.21	1.20	1.14	1.13	1.07	1.06
Digestible lysine, %	1.28	1.27	1.14	1.14	1.08	1.08	1.02	1.01
Digestible methionine ^4^, %	0.63	0.63	0.58	0.58	0.55	0.55	0.52	0.52
Digestible methionine + cysteine, %	0.91	0.91	0.85	0.84	0.81	0.81	0.77	0.77
Arginine to Lysine	1.05	1.05	1.06	1.06	1.05	1.05	1.05	1.05
Sodium, %	0.17	0.17	0.18	0.18	0.18	0.18	0.18	0.18
Potassium, %	0.89	0.88	0.81	0.80	0.76	0.76	0.72	0.72
Chloride, %	0.21	0.21	0.21	0.21	0.21	0.21	0.21	0.21
DCAD ^5^, mEq/kg	242	242	223	222	212	211	202	201

^1^ Aflatoxin B_1_ was detected lower than 8 µg/kg. ^2^ Evonik Nutrition & Care GmbH. ^3^ The premixes provided the following per kilogram of diet: zinc, 88 mg; iron, 16 mg; manganese, 96 mg; copper, 12.8 mg; iodine, 1 mg; selenium, 0.24 mg; vitamin A, 10,000 IU; vitamin D_3_, 4000 IU; vitamin E, 36 mg; vitamin K_3_, 3 mg; thiamine, 3.2 mg; riboflavin, 7.2 mg; pantothenic acid, 12 mg; niacin, 52 mg; pyridoxine, 3.36 mg; folic acid, 2.08 mg; vitamin B_12_, 20 µg; biotin, 120 µg; and choline chloride, 400 mg. ^4^ The excess of calculated methionine converts to cysteine to provide methionine + cysteine. ^5^ Dietary cation–anion difference.

**Table 15:** Analyzed and calculated concentrations of Aflatoxins.

	Moldy Rice Powder ^1^	Diets ^2^
Aflatoxins	mg/kg	µg/kg
B_1_	65.6	500
B_2_	2.1	16
G_1_	25.5	195
G_2_	0.9	7.0
Total	94.1	718

^1^ Analyzed by HPLC. ^2^ The dietary concentration was calculated based on the portions of the contaminated rice (moldy rice powder).

## Data Availability

Raw data sharing is not applicable to this article.

## Supplementary Materials

The following are available online at https://www.mdpi.com/article/10.3390/toxins14030192/s1, Table S1: Interaction effect between L-Threonine and Mycofix Plus (MP); on breast meat yield and LDL^1^ of broilers, Cobb 500^2^; Table S2: Interaction effect between Mycofix Plus (MP) and Aflatoxin B_1_, on Cholesterol, HDL^1^, ALT^2^, and LDH^3^ of broilers, Cobb 500^4^; Table S3: Interaction effect between L-Threonine and Mycofix Plus (MP), on IBV^1^ titer of broilers, Cobb 500^2^; Table S4: Interaction effect between Mycofix Plus (MP) and Aflatoxin B_1_, on IBV^1^ titer of broilers, Cobb 500^2^. Table S5: Effect of L-Threonine and Mycofix Plus (MP) on meat quality of broilers exposed to Aflatoxin B_1_, Cobb 500; Table S6: Effect of L-Threonine and Mycofix Plus (MP) on cecal microflora of broilers exposed to Aflatoxin B_1_, Cobb 500.
