# Supplementary material for: Effect of Dietary L-Threonine and Toxin Binder on Performance, Blood Parameters, and Immune Response of Broilers Exposed to Aflatoxin B1
Source: Toxins (Basel). 2022 Mar 4;14(3):192. doi: 10.3390/toxins14030192 (PMC8951136; doi:10.3390/toxins14030192)
Supplement: Supplementary file 1 [file toxins-14-00192-s001.zip › toxins-1588703-supplementary.pdf]

Effect of Dietary L-Threonine and Toxin Binder on Performance, Blood Parameters, and Immune Response of Broilers Exposed to Aflatoxin B_1

Aydin Mesgar, Habib Aghdam Shahryar, Christopher Anthony Bailey, Yahya Ebrahimnezhad and Anand Mohan

Table S1. Interaction effect between L-Threonine and Mycofix Plus (MP); on breast meat yield and LDL[1] of broilers, Cobb 500[2].

Independent Variables		Breast	LDL
L-Threonine, % of Requirements	MP, g/kg	Relative Weight, % of Live Body Weight	mg/dl
100	0	21.32^{ab}	24.37^{b}
100	1	20.06^{b}	33.88^{ab}
125	0	21.28^{ab}	36.22^{a}
125	1	21.89^{a}	29.59^{ab}
P-value		*	*

^{a,b}Means within a column with differing superscripts are significantly different at $^{*}P < 0.05$. [1]Low-Density Lipoprotein. [2]Means represent 16 pens of chickens with 10 birds per pen (*n* = 16/group).

Table S2. Interaction effect between Mycofix Plus (MP) and Aflatoxin B_1, on Cholesterol, HDL[1], ALT[2], and LDH[3] of broilers, Cobb 500[4].

Independent Variables		Cholesterol	HDL	ALT	LDH
MP, g/kg	Aflatoxin B_1, 500 ppb	mg/dl	mg/dl	u/l	u/l
0	-	131.91^{ab}	80.31^{a}	3.88^{b}	811.00^{ab}
0	+	122.92^{b}	74.31^{b}	5.31^{a}	1074.88^{a}
1	-	124.81^{ab}	77.00^{ab}	4.85^{ab}	936.69^{ab}
1	+	133.29^{a}	78.88^{ab}	4.13^{ab}	760.38^{b}
P-value		*	*	*	*

^{a,b}Means within a column with differing superscripts are significantly different at $^{*}P < 0.05$. [1]High-Density Lipoprotein. [2]Alanine aminotransferase. [3]Lactate dehydrogenase. [4]Means represent 16 pens of chickens with 10 birds per pen (*n* = 16/group).

Table S3. Interaction effect between L-Threonine and Mycofix Plus (MP), on IBV[1] titer of broilers, Cobb 500[2].

Independent Variables		IBV
L-Threonine, % of Requirements	MP, g/kg	Log_{10}
100	0	3.829^{ab}
100	1	3.824^{ab}
125	0	3.823^{b}
125	1	3.830^{a}
P-value		**

^{a,b}Means within a column with differing superscripts are significantly different at $^{**}P < 0.01$. [1]Infectious Bronchitis Virus. [2]Means represent 16 pens of chickens with 10 birds per pen (*n* = 16/group).

Table S4. Interaction effect between Mycofix Plus (MP) and Aflatoxin B_1, on IBV[1] titer of broilers, Cobb 500[2].

Independent Variables		IBV[2]
MP, g/kg	Aflatoxin B_1, ppb	Log_{10}
0	-	3.831[a]
0	+	3.821[b]
1	-	3.826[ab]
1	+	3.827[a]
P-value		**

[a,b]Means within a column with differing superscripts are significantly different at $^{**}P < 0.01$. [1]Infectious Bronchitis Virus. [2]Means represent 16 pens of chickens with 10 birds per pen (n = 16/group).

Table S5. Effect of L-Threonine and Mycofix Plus (MP) on meat quality of broilers exposed to Aflatoxin B_1, Cobb 500.

				pH	WHC[1]	Cook Loss	MDA[2], Breast	MDA, Drumstick
Treatments	L-Threonine, % of Requirements	MP, g/kg	Aflatoxin B_1, 500 ppb		%	%	mg MDA/kg of meat	mg MDA/kg of meat
T_1	100	0	-	5.93	46.12	23.28	1.39	2.46
T_2	100	0	+	5.88	47.06	25.42	1.41	2.28
T_3	100	1	-	5.91	48.80	24.75	1.32	2.23
T_4	100	1	+	5.94	46.93	21.52	1.54	2.38
T_5	125	0	-	5.86	47.66	22.72	1.43	2.36
T_6	125	0	+	5.92	47.47	23.66	1.55	2.46
T_7	125	1	-	5.89	50.91	23.09	1.68	2.34
T_8	125	1	+	5.90	50.85	23.48	1.53	2.29
	Pooled SEM			0.04	2.15	1.37	0.12	0.16
Main Effects			Levels			Means[3]		
L-Threonine			100	5.91	47.23	23.74	1.41	2.34
			125	5.89	49.22	23.24	1.55	2.36
Mycofix Plus			0	5.90	47.08	23.77	1.44	2.39
			1	5.91	49.37	23.21	1.52	2.31
Aflatoxin B_1			-	5.90	48.37	23.46	1.45	2.35
			+	5.91	48.08	23.52	1.51	2.35
Main Effects and Interaction Effects						*P*-values		
L-Threonine				NS	NS	NS	NS	NS
Mycofix Plus				NS	NS	NS	NS	NS
Aflatoxin B_1				NS	NS	NS	NS	NS
L-Threonine × Mycofix Plus				NS	NS	NS	NS	NS
L-Threonine × Aflatoxin B_1				NS	NS	NS	NS	NS
Mycofix Plus × Aflatoxin B_1				NS	NS	NS	NS	NS
L-Threonine × Mycofix Plus × Aflatoxin B_1				NS	NS	NS	NS	NS

[1]Water Holding Capacity. [2]Malondialdehyde. [3]Means represent 32 pens of chickens with 10 birds per pen (n = 32/group). NS, $P \geq 0.05$.

Table S6. Effect of L-Threonine and Mycofix Plus (MP) on cecal microflora of broilers exposed to Aflatoxin B_1, Cobb 500.

				TAC[1]	*E. coli*[2]	*Lactobacilli*
Treatments	L-Threonine, % of Requirements	MP, g/kg	Aflatoxin B_1, 500 ppb	CFU[3]	CFU	CFU
T_1	100	0	-	8.30	8.13	8.02
T_2	100	0	+	8.36	8.20	8.24
T_3	100	1	-	8.37	7.92	8.24
T_4	100	1	+	8.28	8.02	8.05
T_5	125	0	-	8.36	7.96	8.23
T_6	125	0	+	8.34	7.75	8.17
T_7	125	1	-	8.22	8.00	8.26
T_8	125	1	+	8.39	8.05	8.24
Pooled SEM				0.09	0.16	0.11
Main Effects			Levels		Means[4]	
L-Threonine			100	8.33	8.07	8.14
			125	8.32	7.94	8.22
Mycofix Plus			0	8.34	8.01	8.16
			1	8.31	8.00	8.20
Aflatoxin B_1			-	8.31	8.00	8.19
			+	8.34	8.01	8.18
Main Effects and Interaction Effects					*P*-values	
L-Threonine				NS	NS	NS
Mycofix Plus				NS	NS	NS
Aflatoxin B_1				NS	NS	NS
L-Threonine × Mycofix Plus				NS	NS	NS
L-Threonine × Aflatoxin B_1				NS	NS	NS
Mycofix Plus × Aflatoxin B_1				NS	NS	NS
L-Threonine × Mycofix Plus × Aflatoxin B_1				NS	NS	NS

[1]Total Aerobic Bacteria Counts. [2]Escherichia Coli. [3]Colony Forming Unit; Values are log_{10} of colony-forming units of cecal content (CFU) g^{-1}. [4]Means represent 32 pens of chickens with 10 birds per pen (n = 32/group). NS, $P \geq 0.05$.
